# Spinal Cord Injury in Enterovirus D68 Infection: Mechanisms and Pathophysiology in a Mouse Model

**DOI:** 10.3390/v17111478

**Published:** 2025-11-06

**Authors:** Wei Duan, Jichen Li, Ying Liu, Rui Wang, Qian Yang, Huijie Li, Yucai Liang, Qi Shi, Jiao Wang, Jinbo Xiao, Jianfang Zhou, Qiang Sun, Yong Zhang

**Affiliations:** 1 National Key Laboratory of Intelligent Tracking and Forecasting for Infectious Diseases (NITFID), National Institute for Viral Disease Control and Prevention, Chinese Center for Disease Control and Prevention, Beijing 102206, China; duanwei0617@126.com (W.D.); jichenli666@163.com (J.L.); ilene1996@163.com (Y.L.); ruiwang_97@163.com (R.W.); yangqian@ivdc.chinacdc.cn (Q.Y.); lihuijie_jiejie@163.com (H.L.); yucai.liang@hotmail.com (Y.L.); shiqi@ivdc.chinacdc.cn (Q.S.); wangjiao@ivdc.chinacdc.cn (J.W.); mr_mint1114@sina.com (J.X.); zhoujf@ivdc.chinacdc.cn (J.Z.); 2 National Polio Laboratory, National Institute for Viral Disease Control and Prevention, Chinese Center for Disease Control and Prevention, 155 Changbai Road, Beijing 102206, China; 3 National Health Commission Key Laboratory of Microbial Genomics, National Health Commission Key Laboratory of Laboratory Biosafety, National Institute for Viral Disease Control and Prevention, Chinese Center for Disease Control and Prevention, 155 Changbai Road, Beijing 102206, China; 4 World Health Organization Polio Reference Laboratory for the Western Pacific Region, National Institute for Viral Disease Control and Prevention, Chinese Center for Disease Control and Prevention, Beijing 102206, China

**Keywords:** Enterovirus D68, acute flaccid myelitis, mouse model, RNA-seq, mitochondrial dysfunction

## Abstract

Enterovirus D68 (EV-D68) is a significant global pathogen associated with severe respiratory infections and acute flaccid myelitis in children. Currently, there are no vaccines or antiviral drugs available for EV-D68, and a robust model to elucidate the pathogenesis of EV-D68 and evaluate treatment methods is lacking. We developed a mouse-adapted EV-D68 strain that caused progressive limb paralysis after intramuscular inoculation in 7-day-old mice. Viral load analysis showed that the skeletal muscle and spinal cord had the highest titers and most severe injuries. RNA sequencing of the infected muscle, brain, spinal cord, and lung tissues revealed differentially expressed genes (DEGs) associated with viral infection and pathogenesis. DEGs were significantly enriched in various pathways associated with antiviral immunity, interferon responses, and cytokine signaling. In the spinal cord, DEGs highlighted mitochondrial dysfunction and oxidative stress as crucial contributors to neural damage. Flow cytometry analysis of spinal cord cells showed that EV-D68 activates the immune system, leading to systemic inflammation and significant increases in CD8^+^ and CD4^+^ T cells, but limited neutrophil and monocyte infiltration. This mouse model provides a valuable tool for studying EV-D68 pathogenesis and evaluating antiviral and vaccine efficacy, thereby advancing the understanding of its neuropathological mechanisms. **Importance:** We developed a novel mouse model of EV-D68 that provides a valuable tool for studying its pathogenesis and evaluating antiviral and vaccine efficacy, deepening the understanding of its neuropathological basis.

## 1. Introduction

Enterovirus D68 (EV-D68) is a non-enveloped single-stranded RNA virus belonging to the family *Picornaviridae* and the genus *Enterovirus* (species *Enterovirus deconjuncti*, previously named *Enterovirus D*), is a significant pathogen associated with severe respiratory illnesses and acute flaccid myelitis (AFM), particularly in children [1,2]. First isolated in California in 1962, EV-D68 was rarely reported until the early 21st century, with increased detection in North America, Europe, and Asia [3,4]. In 2014, the United States reported 1153 confirmed cases of severe respiratory illness and 14 deaths associated with the EV-D68 outbreak, marking a significant epidemic event [5,6]. Notably, this coincided with a surge of 120 pediatric AFM cases, a rare poliomyelitis-like syndrome characterized by acute limb weakness and spinal cord abnormalities on magnetic resonance imaging [7,8]. The temporal overlap between AFM cases and EV-D68 circulation strongly suggested causality, which was later confirmed by case reports and epidemiological investigations [9,10].

No effective vaccines or antiviral agents are currently available for EV-D68, underscoring the urgent need for animal models to elucidate its pathogenesis and evaluate therapeutic strategies. Blanco et al. have confirmed that the cotton rats serve as a viable model for studying systemic EV-D68 infection, demonstrating that the virus can spread to the central nervous system and cause neurological diseases, thus providing a platform for evaluating antiviral intervention [11]. Similarly, intranasal infection of ferrets with EV-D68 has been shown to induce clinical symptoms [12]. Hixon et al. demonstrated that neonatal Swiss Webster mice infected intramuscularly or intracerebrally with EV-D68 clinical isolates developed AFM-like paralysis, which correlated with spinal motor neuron infection and loss [13]. Mouse models dominate EV-D68 studies, with AG129 mice infected with EV-D68 strains shown to exhibit respiratory diseases and be suitable for antiviral screening. Neonatal mice infected with EV-D68 strains from the 2014 outbreak via intraperitoneal (i.p.), intramuscular (i.m.), or intracerebral (i.c.) routes consistently exhibit AFM and spinal motor neuron degeneration [6,13,14]. Robust animal models are critical for understanding EV-D68 neuropathogenesis, systemic effects, and testing preventive and therapeutic interventions [15,16].

Mitochondria are critical organelles that regulate key cellular processes, including ATP production, generation of reactive oxygen species, calcium dynamics, and apoptosis initiation [17,18]. They play critical roles in antiviral defense mechanisms [19]. However, numerous viruses, including enteroviruses, have evolved strategies to impair mitochondrial function, thereby subverting the host immune response [20]. These impairments manifest in diverse ways, including the induction of mitophagy, disruption of mitochondrial bioenergetics [21], exacerbation of mitochondrial dysfunction, and impairment of energy metabolism and cell survival, particularly in energy-demanding tissues such as the nervous system [22]. In this study, we developed a mouse-adapted EV-D68 strain capable of infecting 7-day-old mice via i.m. inoculation. This model recapitulates notable clinical features of AFM, including progressive limb paralysis and age- and dose-dependent mortality, with viral replication predominantly found in the skeletal muscle and spinal cord. Using RNA sequencing (RNA-seq), we profiled differentially expressed genes (DEGs) in infected tissues to reveal the molecular mechanisms underlying EV-D68-induced neural injury. Our findings highlight mitochondrial dysfunction and oxidative stress as central drivers of spinal cord damage, which advances the understanding of EV-D68 neurotropism and provides a foundation for evaluating therapeutic interventions targeting mitochondrial and immune pathways.

## 2. Materials and Methods

### 2.1. Ethics Statement

All animal experiments were approved by the Ethics Review Committee of the National Institute for Viral Disease Control and Prevention, Chinese Center for Disease Control and Prevention (Approval No. 20201022059). Specific-pathogen-free (SPF)-grade Institute of Cancer Research (ICR) mice were procured from SPF Biotechnology Co., Ltd. (Beijing, China) and maintained under controlled conditions at the Animal Center of the Chinese Center for Disease Control and Prevention. The mice were housed in individually ventilated cages with ad libitum access to food and water. All animal procedures were conducted under strict SPF-grade housing conditions to ensure a pathogen-free environment.

### 2.2. Virus and Cells

EV-D68 clinical isolate strain D2 was obtained from a throat swab of a pediatric patient presenting with fever, cough, and limb weakness in Beijing in 2018. Human rhabdomyosarcoma (RD) cells were cultured in Dulbecco’s modified Eagle’s medium (DMEM) supplemented with 10% fetal bovine serum (FBS; San Diego, California, CA, USA) and 1% penicillin–streptomycin (HyClone, Logan, UT, USA). Cultures were maintained in a humidified incubator at 33 °C with 5% CO_2_. When the cells reached 80–90% confluence, they were exposed to the virus in DMEM containing 2% fetal bovine serum to induce cytopathic effects (CPEs). After three freeze–thaw cycles, the viral suspension was filtered through a 0.22-μm filter and stored at −80 °C until further use.

### 2.3. Animal Infection Experiments

In this study, 7-day-old ICR mice were inoculated with the EV-D68 D2-P9 strain via i.m. injection into the left hindlimb thigh muscle (quadriceps femoris), and daily symptom observations and body weight checks were conducted. To establish an EV-D68 D2 ICR mouse model, we infected 2-day-old ICR mice with the parental EV-D68 D2 strain (50 µL) via i.m. injection. The mice were sacrificed 5 days post-infection, and the spinal cord tissues were collected, homogenized with 300 µL of phosphate-buffered saline (PBS), and centrifuged at 12,000× *g* for 20 min. The supernatant was used to inoculate RD cells to induce CPEs. Once CPEs appeared, the viral suspension was subjected to three freeze–thaw cycles, centrifuged at 12,000× *g* for 20 min, and filtered through a 0.22-μm membrane filter. Subsequently, the supernatant was intramuscularly injected into 2-day-old ICR mice. This process was repeated nine times for each passage (P1 to P9). By the ninth passage (P9), 7-day-old mice exhibited AFM-like symptoms, including limb weakness and other neurological deficits. The concentrated virus was subsequently used to infect ICR mice that exhibited progressive paralysis. The body weight, survival rate, and clinical scores were monitored daily. Increasing clinical scores were correlated with disease severity. The clinical scoring criteria were as follows: 0, no disease; 1, ruffled fur; 2, loss of weight; 3, single-limb paralysis; 4, paralysis of both hindlimbs; and 5, moribund or dead.

### 2.4. Tissue Collection and Viral Titration

On days 1, 3, 5, and 7 after infection, brain, forelimb muscle, hindlimb muscle, heart, liver, kidney, lung, intestine, spleen, and spinal cord tissues were collected from infected mice and control mice. Before collecting tissue samples, we performed mouse cardiac perfusion using a 2 mL syringe to eliminate blood from the tissues. When conducting the viral titration (TCID_50_) assay, we used homogenates from the entire brain and the complete spinal cord, spanning from the cervical to the lumbosacral levels. The entire tissues were homogenized in 500 µL PBS containing 1% penicillin–streptomycin and crushed in a tissue grinder (Scientz, Ningbo, China), After undergoing three freeze–thaw cycles, the tissues were centrifuged to obtain the supernatant. A total of 50 µL of the supernatant was serially diluted 10-fold and inoculated into RD cells in 96-well plates. After continuous observation for seven days, the viral titer was determined using the TCID_50_ assay. The viral TCID_50_ was calculated using the Reed-Muench method.

### 2.5. Histopathological and Immunofluorescence Assays

At 5 dpi, tissues, including the brain, spinal cord, lungs, forelimb muscles, and hindlimb muscles, were collected from the experimental and control groups of 7-day-old ICR mice that had been injected intramuscularly with the EV-D68 D2-P9 strain. The tissues were fixed in formalin buffer for 24 h, dehydrated, embedded in paraffin, and sectioned into 5-μm-thick slices. We carefully selected specific sections to ensure thorough examination: Brain tissue: we analyzed sagittal sections of the entire brain. This section plane allows for simultaneous examination of multiple key regions, including the cerebral cortex, corpus callosum, hippocampus, thalamus, hypothalamus, cerebellum, and medulla oblongata; Spinal cord tissue: given the prominent EV-D68-related pathological manifestations in the spinal cord, we focused detailed histological examination on the transverse sections of the lumbosacral spinal cord, as this region is often severely affected. The tissue sections were deparaffinized and stained with hematoxylin and eosin. For the immunohistochemistry (IHC) experiment, the brain, spinal cord, lungs, and muscle tissues were dewaxed, dehydrated, and microwaved for 10 min for antigen repair. After blocking with 3% bovine serum albumin, the sections were incubated with rabbit polyclonal anti-EV-D68 VP1 antibody (1:100 dilution; GeneTex, Irvine, TX, USA). Secondary horseradish peroxidase-conjugated antibodies (1:1000 dilution) were added, and the mixture was incubated for 50 min at room temperature. Furthermore, 3,3′-diaminobenzidine and hematoxylin were used for visualization and counterstaining, respectively. K-Viewer-1.7.1.1 or Case Viewer software (version: 2.4.0.119028) was used to analyze all images at magnifications of ×100 and ×400.

### 2.6. Preparation of Single Cell Suspensions

For brain and spinal cord single cell preparation, brain and spinal cord tissues were removed and digested with 2 µg/mL of collagenase II (Biosharp, Beijing, China) at 37 °C for 30 min, followed by mixing with EDTA (0.5 M) (Beyotime, Shanghai, China) for 5 min. Tissues were filtered through a 40-μm cell strainer and resuspended in 40% Percoll (Biosharp, BS909-100 mL) for centrifugation at 1400× *g* for 20 min, before collecting the cell pellets for further analysis. The lung was removed and digested with 1 mg/mL of collagenase IV (Biosharp, BS165-1g) at 37 °C for 60 min, followed by 5 min of mixing with 0.5 M EDTA. Tissues were filtered through a 40-μm cell strainer and resuspended in 30% Percoll for centrifugation at 1400× *g* for 20 min. Cell pellets were collected and lysed in a lysing solution containing NH4Cl, NaHCO_3_, and EDTA. Splenocytes were isolated and digested with 1 mg/mL of collagenase IV at 37 °C for 30 min, followed by 5 min of mixing with 0.5 M EDTA. Tissues were filtered through a 40-μm cell strainer. Subsequently, erythrocytes were lysed, and the pelleted cells were centrifuged. Cells were suspended in 1 mL of PBS, counted, and diluted to the desired volume for later use.

### 2.7. Flow Cytometry Analysis

Seven-day-old ICR mice were intramuscularly injected with 10^9^ TCID_50_ of the EV-D68 D2-P9 strain. At 5 dpi, D2-P9- and mock-infected mice (*n* = 3 per group) were euthanized, and single cells from the brain, spleen, lungs, and spinal cord were extracted for staining. The antibodies included APC/Cyanine7 anti-mouse CD45 (#147718), FITC anti-mouse CD45 (#103108), FITC anti-mouse/human CD11b (#101206), APC anti-mouse Ly-6G (#127614), PerCP/Cy5.5 anti-mouse Ly-6C (#128012), APC/Cy7 anti-mouse CD4 (#100526), and APC Rat anti-mouse CD8a (#553035) (BioLegend Inc., San Diego, CA, USA) were used in the flow cytometry analysis. Dead cells were excluded using the Zombie Violet Fixable Viability Kit (BioLegend Inc., San Diego, CA, USA). Flow cytometry was performed using the BECKMAN COULTER CytExpert SRT system. Detailed flow cytometry gating strategy is depicted in Supplementary Figure S1 and the data were analyzed using its built-in software. The experiment was independently repeated three times.

### 2.8. Construction of a Transcriptome Sequencing Library

Seven-day-old ICR mice (*n* = 3 per group) were inoculated with 10^9^ TCID50 of the EV-D68 D2-P9, while the control group received an equivalent volume of uninfected culture medium. Total RNA was extracted from the brain, spinal cord, skeletal muscle, and lungs of mice using TRIzol reagent. The RNA quality and integrity were assessed, and samples with an RNA integrity number of >7 were used for subsequent library preparation. Briefly, the first strand of complementary DNA (cDNA) was synthesized using random primers and reverse transcription, followed by the synthesis of the second cDNA strand using RNase H and DNA polymerase. The enriched and purified cDNA libraries were sequenced using an Illumina NovaSeq 6000 platform. We have deposited the raw RNA-seq data generated in this study into the National Genomics Data Center (NGDC) database under accession number PRJCA048578.

### 2.9. RNA-Seq Data Bioinformatics Analysis

Differential gene expression analysis was conducted using DESeq2 software (version: 1.49.4), with significance thresholds set at *p* < 0.05 and |log_2_FC| > 1.5 for DEG identification. To elucidate the biological significance of DEGs, Gene Ontology (GO) and Kyoto Encyclopedia of Genes and Genomes (KEGG) pathway analyses were performed using standard approaches. The GO analysis focused on three primary categories, namely cellular component (CC), biological process (BP), and molecular function (MF). KEGG pathway analysis was used to correlate DEGs with higher-level biological systems, such as cellular processes, organismal systems, and ecosystems, to gain insights into molecular response networks involving protein-coding genes. Enrichment analysis was performed using the Database for Annotation, Visualization, and Integrated Discovery Bioinformatics Resources 6.8.

### 2.10. Statistical Analysis

Statistical analyses were performed using GraphPad Prism 8.0. Data are presented as the mean ± SD, with each experiment conducted independently to ensure robust findings. Survival differences among mouse cohorts were analyzed using the log-rank (Mantel–Cox) test. Variations in tissue viral titers across mouse groups were assessed using a one- or two-way analysis of variance. Statistical significance was defined as *p* < 0.05, with the following notations: ** *p* < 0.01, *** *p* < 0.001, **** *p* < 0.0001, and “ns” indicating no significant difference.

## 3. Results

### 3.1. Development of an EV-D68 Infection Mouse Model

**Figure 1:**
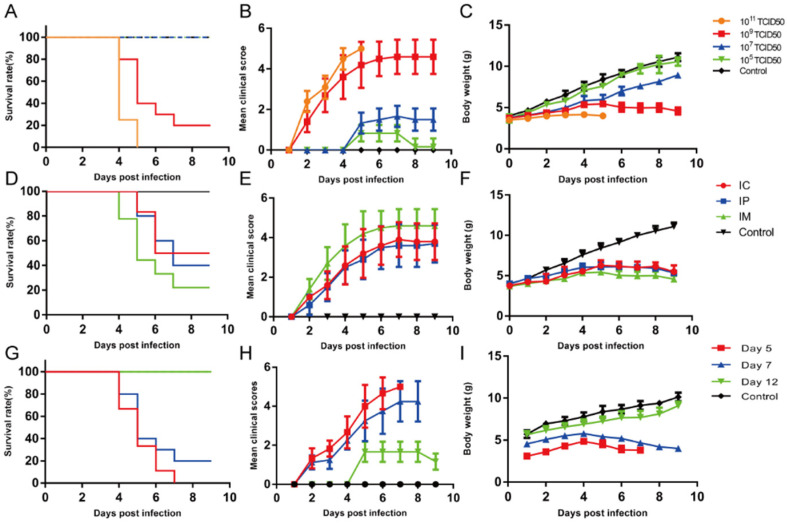
Establishment of EV-D68-infected mouse models. Seven-day-old ICR mice were infected with EV-D68 D2-P9 at doses of 10^5^–10^11^ TCID_50_ via i.m. injection (*n* = 6 per group); control mice were inoculated with uninfected culture medium. The survival rates, clinical scores, and body weight changes were monitored daily (**A**–**C**). Institute of Cancer Research (ICR) mice were infected with 10^9^ TCID_50_ of EV-D68 D2-P9 via i.c., i.p., or i.m., with survival rates, clinical scores, and body weight changes recorded daily (**D**–**F**). ICR mice aged 5, 7, or 12 days were infected with 10^9^ TCID_50_ of EV-D68 via the i.m. route, and survival rates, clinical scores, and body weight changes were assessed daily (**G**–**I**). The experiment was independently repeated three times.

To elucidate the pathogenesis of EV-D68-induced AFM, we developed a mouse-adapted EV-D68 strain (EV-D68 D2-P9) that caused progressive limb paralysis in ICR mice after i.m. inoculation. Briefly, 7-day-old ICR mice were inoculated i.m. with EV-D68 D2-P9 at doses ranging from 10^5^ to 10^11^ TCID_50_, and the clinical manifestations were assessed daily. At a dose of 10^11^ TCID_50_/mL, all mice died within 5 dpi, exhibiting severe hindlimb paralysis and progressive weight loss. When the dose was reduced to 10^9^ TCID_50_/mL, the mortality rate reached 80% at 7 dpi, followed by no deaths; the surviving mice showed significant hindlimb paralysis and gradual weight loss. Doses ranging from 10^5^ to 10^7^ TCID_50_/mL caused mild lower limb motor dysfunction, with no deaths or significant weight reduction (Figure 1A–C). We further evaluated the influence of the administration route by inoculating 7-day-old ICR mice with 10^9^ TCID_50_ of EV-D68 D2-P9 via i.m., i.c., or i.p. routes, with survival rates of 50%, 40%, and 20%, respectively, at 7 dpi, with clinical scores ranging from 3 to 5, indicating moderate-to-severe nerve damage, with gradual weight loss (Figure 1D–F). We observed the clinical symptoms of mice after different routes of virus attack and found that mice exhibited progressive paralysis of one or both lower limbs and forelimbs after i.m. injection, whereas no such symptom progression was observed after intracranial and abdominal injections. Multiple studies have also reported that, among various routes of infection in mice, such as intracranial, nasal, intraperitoneal, and i.m. injection, the incidence rate is highest through the i.m. route, while it is lower through the nasal and intraperitoneal routes [13]. Age-dependent susceptibility was assessed by i.m. inoculation of 10^9^ TCID_50_ in 5-, 7-, or 12-day-old mice. The 5- and 7-day-old mice exhibited severe paralysis and mortality by 5–8 dpi, with significant weight loss, closely mimicking human AFM. In contrast, 12-day-old mice exhibited mild hindlimb motor dysfunction with 100% survival (Figure 1G–I). The control mice remained asymptomatic. These results indicate that the mouse-adapted EV-D68 strain (EV-D68 D2-P9) could successfully infect 7-day-old ICR mice and induce AFM symptoms.

### 3.2. Effective Replication of EV-D68 D2-P9 in Mouse Tissues

**Figure 2:**
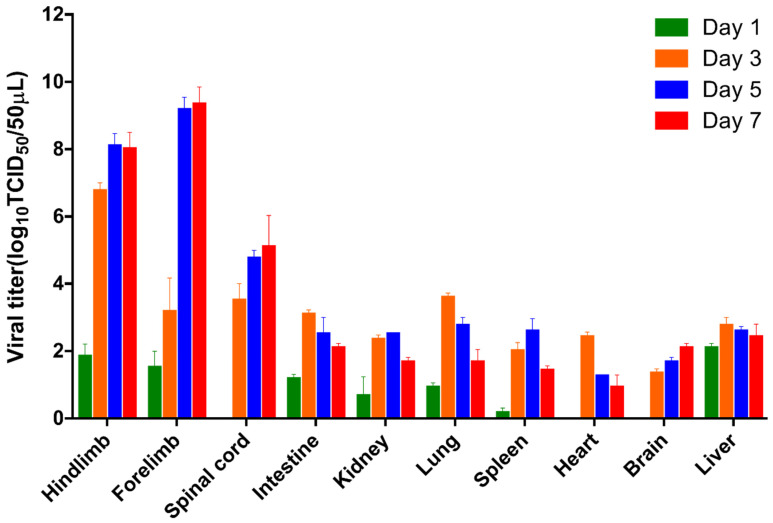
Virus titers in tissues of mice infected with EV-D68 D2-P9. Seven-day-old ICR mice were infected with EV-D68 D2-P9 via intramuscular injection, and the viral titers in different tissues (hindlimb muscle, forelimb muscle, spinal cord, intestine, kidney, lung, spleen, heart, brain, and liver) were measured at 1, 3, 5, and 7 dpi. Data are presented as the mean ± SD from three mice per group. The experiment was independently repeated three times.

To investigate the replication kinetics of EV-D68 D2-P9 in different mouse organs, we administered an i.m. injection of EV-D68 D2-P9 at a dose of 10^9^ TCID_50_ to 7-day-old ICR mice. The virus titers were quantified on days 1, 3, 5, and 7 post-inoculation. The viral titers were significantly higher in the skeletal muscle and spinal cord at 5–7 dpi than in the other tissues (Figure 2). The kidney, heart, lung, spleen, brain, liver, and intestine showed lower titers, peaking at 3 dpi and declining by 7 dpi (Figure 2). No replication occurred in wild-type mice. These findings suggest that the EV-D68 D2-P9 strain displays strong tropism for the skeletal muscle and spinal cord in ICR mice.

### 3.3. Histopathology and Distribution of EV-D68 Antigen

**Figure 3:**
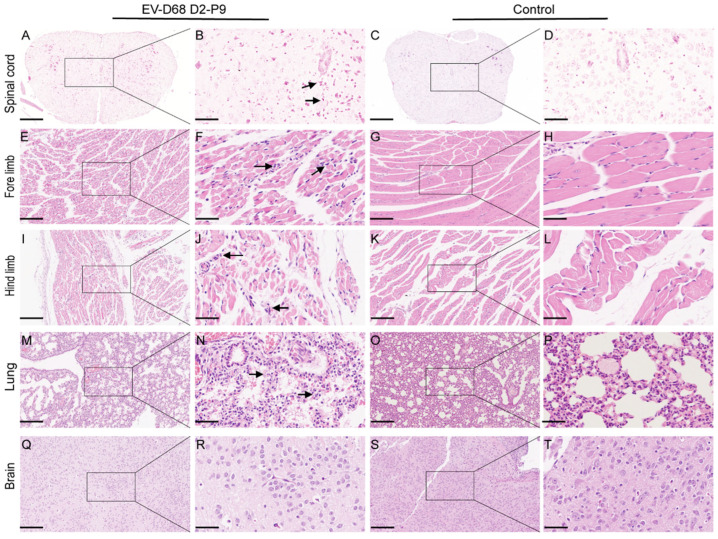
EV-D68 infection leads to severe histopathological changes in multiple organs of neonatal mice. Seven-day-old ICD mice were inoculated with EV-D68 D2-P9 strain via intramuscular injection or mock infection as controls. Tissues were collected for hematoxylin and eosin (H&E) staining five days post-infection (dpi). The spinal cord of EV-D68-infected mice showed extensive inflammatory cell infiltration (black arrows) (**A**,**B**). The spinal cord of mock-infected control mice exhibited normal structure (**C**,**D**). Muscle fibers in the forelimb skeletal muscle of infected mice exhibits necrosis, breakage (arrows), and immune cell infiltration (**E**,**F**). No pathological changes were observed in the control group (**G**,**H**). Muscle fibers in the hind limb skeletal muscle of infected mice exhibits necrosis, breakage (arrows), and immune cell infiltration (**I**,**J**). No pathological changes were observed in the control group (**K**,**L**). Lung tissue of the infected group showed significant thickening of alveolar walls (interstitial pneumonia) and inflammatory exudate in alveolar cavities (arrows) (**M**,**N**). No pathological changes were observed in the control group (**O**,**P**). Mild pathological changes were observed in the brain of infected mice (**Q**,**R**). No pathological changes were observed in the control group (**S**,**T**). Scale bar: 200 µm (**A**,**C**,**E**,**G**,**I**,**K**,**M**,**O**); 50 µm (**B**,**D**,**F**,**H**,**J**,**L**,**N**,**P**).

**Figure 4:**
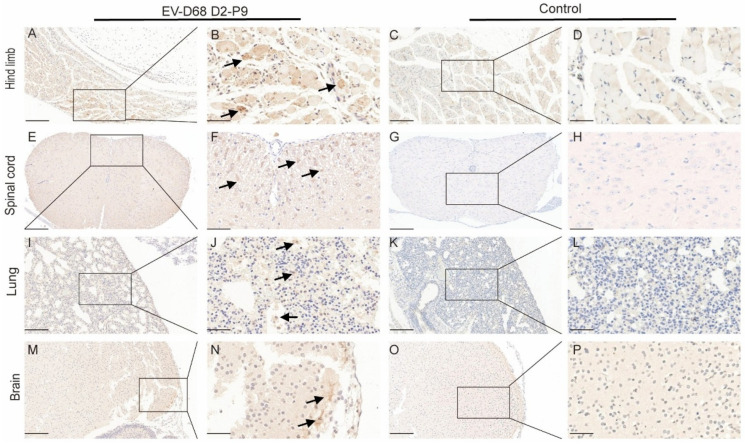
Immunohistochemical detection of the distribution of EV-D68 antigen in multiple tissues of infected mice. After intramuscular inoculation with EV-D68 D2-P9 in 7-day-old mice, IHC was employed to analyze the distribution of viral antigens (arrows) in the hindlimb skeletal muscle (**A**–**D**), spinal cord (**E**–**H**), lung (**I**–**L**), and brain (**M**–**P**) at 5 dpi. Magnifications: (**A**,**C**,**E**,**G**,**I**,**K**,**M**,**O**), Scale bars: 200 µm; other panels, Scale bars: 50 µm.

To investigate the pathogenic mechanisms of the EV-D68 D2-P9 strain, 7-day-old ICR mice were infected via i.m. injection. By 5 dpi, mice exhibited progressive weight loss and neurological symptoms including limb weakness, paralysis, and mortality, consistent with AFM. Histopathological analysis of key tissues revealed severe spinal cord pathology, characterized by anterior horn motor neuron degeneration, nuclear pyknosis, cytoplasmic vacuolation, and white matter axonal degeneration with myelin sheath vacuolation and fragmentation (Figure 3A–D). Skeletal muscles (forelimb and hindlimb) displayed extensive fiber necrosis, fragmentation, inflammatory cell infiltration, and interstitial fibrosis, indicating direct viral impact on muscle integrity (Figure 3E–L). Lung tissue showed interstitial pneumonia, with thickened alveolar walls, edema, emphysema, and inflammatory infiltrates. These findings suggest an inflammatory response and tissue damage in the respiratory system (Figure 3M–P). No significant pathological changes were observed in the brain, suggesting that the brain may be less susceptible to direct viral infection or that infection does not cause severe tissue damage within the observed timeframe (Figure 3Q–T). Immunohistochemistry using an EV-D68 VP1-specific antibody confirmed widespread viral antigen distribution. Intense brown staining was observed in the muscle fiber cytoplasm, indicating active viral replication (Figure 4A–D), and in spinal cord motor neurons, highlighting significant neurotropism (Figure 4E–H). Focal antigen positivity was detected in lung alveolar epithelial cells, with limited staining in inflammatory cells (Figure 4I–L). Brain tissue showed minimal pathology, with only sparse EV-D68 antigen staining in the neuronal cytoplasm, together with mild degenerative changes, suggesting lower susceptibility to severe viral damage (Figure 4M–P). These results indicate that EV-D68 infection in ICR mice induces multiorgan pathological damage and neurological symptoms consistent with human disease, validating this model as a valuable tool for studying EV-D68 pathogenic mechanisms.

### 3.4. EV-D68 D2-P9 Induces Immune System Activation

**Figure 5:**
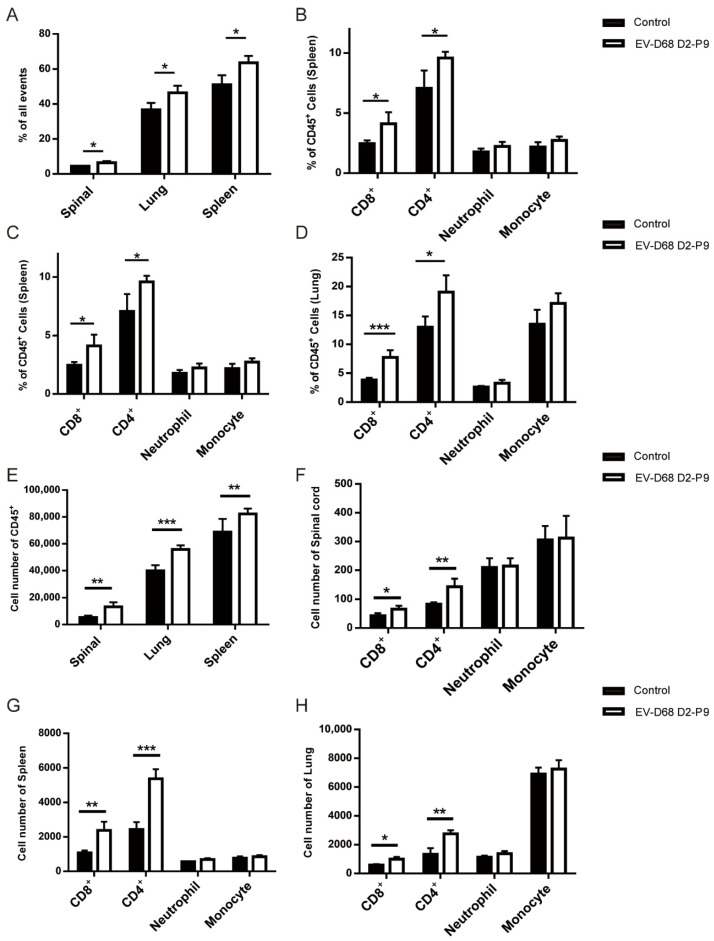
Immune cell infiltration in EV-D68 D2-P9-infected ICR mice. Flow cytometry analysis of CD45^+^ leukocytes (total leukocytes) in the spleen, spinal cord, and lung tissues from 7-day-old ICR mice infected with the EV-D68 D2-P9 strain, compared to mock-infected controls (*n* = 9). (**A**) Proportions of immune cells in the spinal cord, spleen, and lung; (**B**–**D**) The absolute counts of immune cells in the spinal cord, spleen, and lung; (**E**–**H**). The significance of the data was determined by the two-tailed Student’s *t*-tests. * *p* < 0.05; ** *p* < 0.01; *** *p* < 0.001. The experiment was independently repeated three times. Data are represented as mean ± SEM (9 mice per group).

To investigate immune cell infiltration during EV-D68 infection, 7-day-old ICR mice were intramuscularly inoculated with the EV-D68 D2-P9 strain. At 5 dpi, spleen, spinal cord, and lung tissues from infected and mock-infected mice were analyzed via flow cytometry for CD45^+^ leukocytes, CD8^+^ T cells, CD4^+^ T cells, neutrophils (Ly-6G^+^), and monocytes (Ly-6C^+^). Infection with EV-D68 D2-P9 increased the proportion of CD45^+^ leukocytes in the spleen, spinal cord, and lungs (Figure 5A). In the spinal cord, T cells (particularly CD8^+^ and CD4^+^ T cells) significantly increase, While the infiltration of neutrophils and monocytes is relatively limited, indicating that the spinal cord primarily relies on T cell immune responses to combat the virus (Figure 5B). The proportions of CD4^+^ and CD8^+^ T cells in the spleen and lungs also significantly increased, with statistical significance. At the same time, the numbers of neutrophils and monocytes also rose, but without statistical significance (Figure 5C,D), possibly reflecting inflammatory responses and cytotoxic T cell-mediated viral clearance in EV-D68 primary target organs. The absolute number of T cells in the corresponding tissues also increased, further confirming this T cell activation pattern (Figure 5E–H). These findings reveal that EV-D68 D2-P9 infection triggers robust immune activation, potentially causing systemic inflammatory responses.

### 3.5. EV-D68 Infection Induces Enrichment of Mitochondrial Dysfunction and Oxidative Stress-Related Genes in the Spinal Cord at the Transcriptome Level

**Figure 6:**
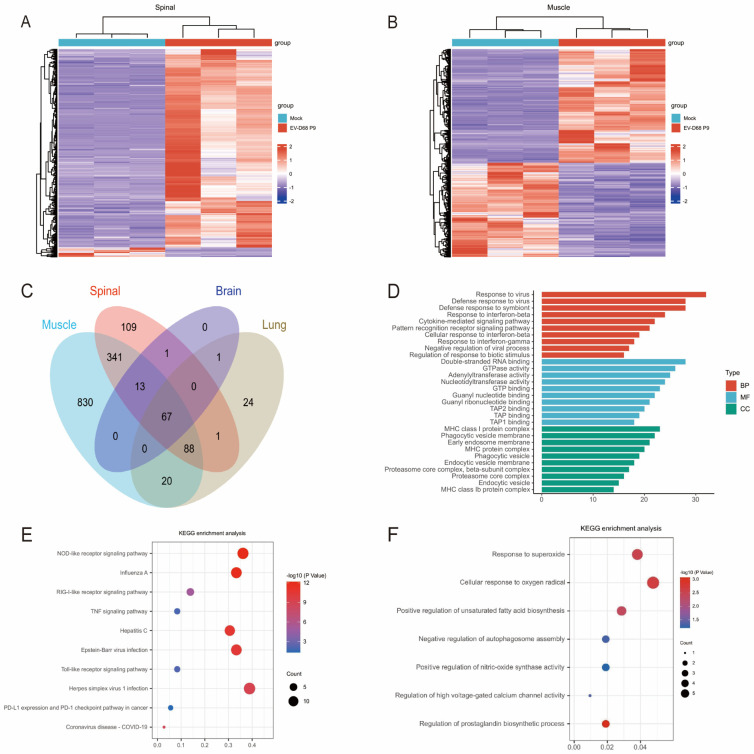
Transcriptomic analysis of EV-D68 D2-P9 strain infection in ICR mice. Differential gene expression analysis in tissues from 7-day-old ICR mice infected with the EV-D68 D2-P9 strain at 5 dpi. RNA-seq was performed on spinal cord, brain, lung, and hindlimb skeletal muscle tissues. The heatmap shows the gene expression differences in muscle tissue and spinal cord (**A**,**B**). Venn diagram illustrating the overlap of upregulated genes across the four tissues (**C**). Functional enrichment analysis of the 67 commonly upregulated genes using GO and KEGG pathways (**D**,**E**). Comparative analysis of spinal cord-specific DEGs highlights unique pathways (**F**).

To investigate the pathological mechanisms of EV-D68 infection, we infected 7-day-old ICR mice with the EV-D68 D2-P9 strain and performed transcriptomic analysis on spinal cord, brain, lung, and hindlimb muscle tissues at 5 dpi. DEGs were identified using |log_2_FC| > 1.5 and adjusted *p* < 0.05. The spinal cord exhibited 621 upregulated and six downregulated genes, the muscle tissue showed 1360 upregulated and 1019 downregulated genes, the brain exhibited 82 upregulated genes, and the lungs displayed 201 upregulated genes (Figure 6A,B). A Venn diagram revealed 67 commonly upregulated genes across all tissues, including *Mx1*, *Irgm1*, *Ifit1*, *Stat1*, *Cxcl10*, *Irf7*, *Tap1*, *Zbp1*, *Oas1g*, *Gbp2*, *Oas1a*, *Nlrc5*, *Ifih1*, *H2-K1*, and *Bst2* (Figure 6C). These genes may be critical mediators of the response to viruses and linked to the antiviral defense mechanisms shared across these tissues. GO analysis revealed significant enrichment in biological processes related to response to virus, defense response to virus, defense response to symbiont, and response to interferon, among others. Cellular component terms such as MHC class I protein complex, phagocytic vesicle membrane, early endosome membrane, and endocytic vesicle membrane were prominent. Molecular functions terms were enriched for double-stranded RNA binding and GTPase activity. KEGG pathway analysis indicated that these genes were involved in viral infections (influenza, measles, coronavirus, hepatitis B/C, and HIV); immune signaling pathways, such as NOD-like, RIG-I-like, Toll-like receptor, and TNF signaling pathways, suggesting strong immune activation; and immune checkpoint modulation through PD-L1/PD-1 interaction (Figure 6D,E). Spinal cord-specific upregulated DEGs were enriched in pathways including response to superoxide, response to oxygen radicals, regulation of unsaturated fatty acid biosynthetic processes, regulation of autophagosome assembly, positive regulation of nitric oxide synthase activity, regulation of high-voltage-gated calcium channel activity, and regulation of prostaglandin biosynthetic processes (Figure 6F). These pathways are closely associated with mitochondrial dysfunction and oxidative stress, with implicated genes including *Hvcn1*, *Gch1*, *Cd36*, *Ptgs2*, *Anxa1*, *Nupr1*, and *Gem*. These results highlight mitochondrial dysfunction and oxidative stress as critical drivers of spinal cord injury and AFM in EV-D68 infection, deepening insights into the neuropathological mechanisms underlying this condition.

## 4. Discussion

EV-D68 is a significant contributor to severe respiratory illnesses and neurological conditions, particularly AFM [23,24]. The development of reliable animal models is essential for studying EV-D68 pathogenesis and evaluating vaccines and antiviral therapies. In this study, we developed a mouse-adapted EV-D68 strain, designated EV-D68 D2-P9, and established an ICR mouse model that closely mimics human AFM, exhibiting progressive limb paralysis and high rates of mortality following i.m. injection. Initial experiments with the parental EV-D68 strain failed to induce paralysis in ICR mice, unlike prior models [6,13,14]. We developed EV-D68 D2-P9 through serial passaging. Following challenge with 10^9^ TCID_50_/mL of virus, 7-day-old mice exhibited weight loss, limb paralysis, and mortality, whereas 12-day-old mice showed milder motor deficits (clinical scores 1–2) without mortality, consistent with the findings of Zhang et al. [6]. Viral titer analysis showed that EV-D68 D2-P9 replicates efficiently in skeletal muscle and spinal cord, peaking at 5–7 dpi, with titers 1–2 orders of magnitude higher than in other tissues, confirming its tropism for these sites. Extensive neuronal damage in spinal cord anterior horn motor neurons, accompanied by axonal degeneration and demyelination, aligns with prior reports on EV-D68 neuropathology [13,25]. To characterize the genomic sequences changes in EV-D68 that cause flaccid paralysis phenotype in infected ICR mice, we conducted whole-genome sequencing on the parental (P0) and adapted (P9) virus strains. (NMDCN00099I4). To understand the molecular changes during the adaptation process of mice, we have provided a detailed analysis of the nucleotide and amino acid substitutions between the parental human isolate (P0) and the final mouse-adapted strain (P9) in Supplementary Table S1. The sequencing results revealed nine nucleotide differences between P0 and P9: VP2-C163A, VP3-T317C, VP3-A699G, VP3-T974A, VP1-A270G, 2B-T247C, 2C-C634T, 3C-T155C and 3D-C945T. Among them, four nucleotide changes resulted in amino acid changes: VP2-Q55K, VP3-R234G, VP1-N90K, and 3C-V52A. These amino acid changes may play a role in inducting symptoms similar to AFM in the ICR mouse model, and further research is needed. Phylogenetic analysis confirms that both the parental and mouse-adapted viruses belong to the EV-D68 D2 evolutionary clade. To elaborate on our findings within the established research context, we note that several key mouse models have aided in our deep understanding of the pathogenesis of EV-D68 prior to our work. These models can be broadly categorized based on their primary research focus. The first category of models aims to simulate natural respiratory infection and systemic spread. For instance, Evans et al., established a model where an EV-D68 strain adapted to mice was inoculated intranasally into AG129 mice, successfully simulating a respiratory disease accompanied by viremia and viral spread to multiple organs, including the CNS [26]. This model is highly valuable for studying the entire disease process (from onset to spread), and evaluating interventions targeting the respiratory phase. However, its reliance on immunodeficient mice and the lack of a consistent and robust paralytic phenotype limit its application in specific research on the mechanisms of AFM. Similarly, Morrey et al. reported that intranasal infection of neonatal IFN-α/β/γ R^-/-^ mice can lead to forelimb paralysis in some animals, but it is primarily associated with myositis rather than severe spinal cord infection [27], highlighting the variability of neurological outcomes. The second category of models has been optimized to reliably induce paralytic myelitis for studying AFM. The pioneering work of Hixon et al. showed that contemporary EV-D68 strains can cause AFM-like paralysis and motor neuron loss in neonatal mice through multiple routes, with intramuscular inoculation being the most effective [13]. Our model aligns with this category and offers unique advantages: Firstly, we use immunocompetent ICR mice, distinct from immunodeficient models (AG129 or IFN-α/β/γ R^-/-^), allowing us to study the role of a complete and normal immune system in pathogenesis and protection. Secondly, our mouse-adapted EV-D68 D2-P9 strain exhibits high neurovirulence across a broader age window of neonatal mice (2-, 5-, and 7-day-old), enhancing experimental flexibility and reproducibility. Additionally, our study provides comprehensive transcriptomic and immunomic analysis of spinal cord infection, offering deeper mechanistic insights into mitochondrial dysfunction, oxidative stress, and T-cell mediated immunopathology in AFM. A recognized limitation of our model, which is also shared by other potent neuropathogenesis models including Hixon’s model, is that it employs intramuscular inoculation, bypassing the natural respiratory tract route. Therefore, our model has advantages in dissecting the core mechanisms of neuronal damage and in high-throughput screening for antiviral drug and vaccine efficacy against neurological disease. It can serve as a complementary tool to respiratory tract models to fully understand the pathogenic mechanisms of EV-D68.

VP1 IHC revealed intense viral presence in spinal cord motor neurons and hindlimb muscle fibers, suggesting that the skeletal muscle is a primary replication site, with potential viral dissemination to the spinal cord via retrograde axonal transport. Limited VP1 staining in brain neurons and lung alveolar epithelial cells indicates tissue-specific tropism, likely influenced by receptor expression and immune responses. We further demonstrated that EV-D68 D2-P9 infection elicits robust immune activation across multiple tissues, characterized by distinct immune cell infiltration patterns in the spleen, spinal cord, and lungs. These results are consistent with previous reports on EV-D68 pathogenesis and host immune responses, such as the findings of Acevedo et al. on IL-52-driven immune cell infiltration [28] and extended observations of macrophages and CD8^+^ T cells in autopsy spinal cord sections from children with flaccid paralysis [29,30]. The significant increase in CD45^+^ leukocytes across the examined tissues highlights the systemic nature of the immune response to EV-D68 D2-P9 infection, particularly in immunocompetent hosts [28]. Notably, the spinal cord exhibited a significant CD8^+^ and CD4^+^ T cell infiltration, consistent with findings of other neurotropic enteroviruses, such as EV-A71 [31]. The immune response characteristics of the spleen and lungs are characterized by a significant increase in the proportion of CD4^+^ and CD8^+^ T cells. In contrast, although the proportion of neutrophils and monocytes has increased, it has not reached a statistically significant level. Our findings establish a link between EV-D68 D2-P9-induced immune activation and the development of systemic inflammatory responses.

In this study, transcriptomic analysis of EV-D68-infected tissues revealed 67 core genes that were consistently upregulated in the spinal cord, brain, lung, and muscle tissues, including *Mx1*, *Irgm1*, *Ifit1*, *Stat1*, and *Cxcl10*, highlighting a conserved antiviral defense program involving interferon signaling, viral RNA sensing (e.g., RIG-I-like receptors), and antigen presentation pathways. This shared response is consistent with established enteroviral immune mechanisms [32]. Subsequently, we observed significant enrichment of pathways related to mitochondrial processes, including regulation of mitochondrion organization, cellular response to oxidative stress, regulation of autophagy, regulation of mitochondrial depolarization, as well as related genes such as *Pmaip1*, *Hgf*, *Bid*, *Plaur*, *Mllt11*, *Mgarp*, *Bak1*, *Psmd10*, *Arrb2*, *Slc7a11*, *Ankrd2*, *Cyp1b1*, *Tlr4*, *Mmp3*, *Aldh3b1*, and *Irgm1*. These pathways and genes are ubiquitous in various tissues (muscle, spinal cord, lung, and brain). After filtering out these common pathways and genes, we further analyzed several biological processes and pathways specifically enriched in the spinal cord after EV-D68 infection in the RNA-seq data. The spinal cord-specific transcriptome analysis revealed distinct enrichments in mononuclear/lymphocyte proliferation and adaptive immune responses, consistent with the histopathological observations of inflammatory infiltration in AFM. Notably, spinal cord-enriched pathways linked to the hyperoxide response, autophagosome regulation, and prostaglandin biosynthesis, mediated by genes such as *Hvcn1, Ptgs2*, and *Trim12c*, provide mechanistic insights into viral neuropathogenesis. The convergence of these pathways strongly suggests mitochondrial dysfunction and oxidative stress, both of which are known drivers of neuronal apoptosis. This is consistent with findings in other enteroviruses (e.g., coxsackievirus B3), which disrupt mitochondrial dynamics, impair energy metabolism, and promote cell death in energy-demanding tissues such as the nervous system [33,34]. Furthermore, EV-D68 infection activated multiple immune signaling pathways, including the *NOD-*, *RIG-I-*, and *Toll*-like receptor pathways, which are integral to host antiviral defense. However, as observed in other enteroviruses, the virus appears to exploit these pathways for immune evasion, potentially through cleavage of mitochondrial antiviral signaling protein, a crucial mediator of Type I interferon responses, as observed in other enteroviruses [35].

Our study has found that after EV-D68 infection, there is significant infiltration of CD4^+^ and CD8^+^ T cells in the spinal cord, accompanied by strong transcriptomic evidence of oxidative stress and mitochondrial dysfunction. Although these findings associate adaptive immunity and neuronal stress with the pathogenesis, they do not explicitly point out a causal relationship. The work of Woods Acevedo et al. provides Key mechanistic insights [28]. Their demonstration that despite comparable spinal cord viral titers, the degree of paralysis was significantly reduced in Ccr2^-/-^ and Rag1^-/-^ mice, or after depleting CD4^+^ or CD8^+^ T cells in wild-type mice, providing compelling evidence that the immune response itself is a main driver of paralytic. Our mice model reveals the presence of high viral titers in the spinal cord, indicating that both direct viral invasion and immune-mediated damage may coexist and potentially synergize. Consequently, the mitochondrial dysfunction and oxidative stress we observed could be the downstream molecular consequences of T cell-mediated cytotoxicity on infected neurons. Alternatively, these pathways may initially be triggered by direct viral infection of motor neurons, with subsequent inflammatory responses amplifying the initial damage. The precise sequence of events and the relative contributions of each mechanism remain to be fully elucidated. Future studies employing similar cell depletion or cytokine blockade strategies in our model are crucial for clarifying these complex interactions. Nonetheless, our findings align with those of Woods Acevedo et al., emphasizing that therapeutic strategies targeting EV-D68-AFM may need to be dual-pronged, simultaneously targeting viral replication and harmful host immune response.

## Data Availability

Data are contained within the article and Supplementary Materials.

## Supplementary Materials

The following supporting information can be downloaded at: https://www.mdpi.com/article/10.3390/v17111478/s1. Figure S1. Gating strategy for flow cytometry analysis. Table S1 Nucleotide and amino acid variation sites in the EV-D68 parental strain and mouse-adapted strains.
