# Supplementary material for: Spinal Cord Injury in Enterovirus D68 Infection: Mechanisms and Pathophysiology in a Mouse Model
Source: Viruses. 2025 Nov 6;17(11):1478. doi: 10.3390/v17111478 (PMC12656879; doi:10.3390/v17111478)
Supplement: Supplementary file 1 [file viruses-17-01478-s001.zip › viruses-3915480-supplementary.pdf]

Supplemental Figure S1

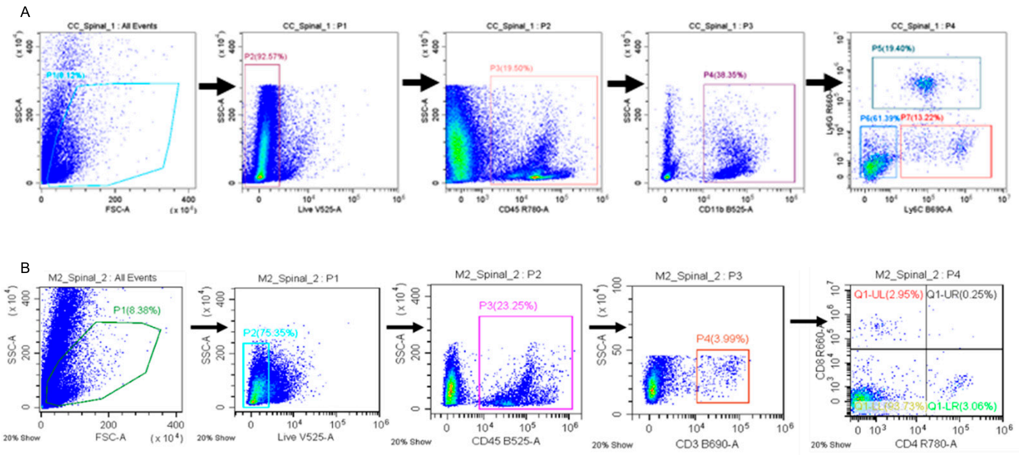

Supplementary Figure S1. Gating strategy for flow cytometry analysis.

The sequential gating strategy used to identify major immune cell populations in the tissues of EV-D68-infected mice is shown. Live cells were first selected, followed by the gating of $CD45^+$ leukocytes. These leukocytes were then subdivided through two parallel analysis strategies: (A) Identification of $CD11b^+$ myeloid cells, which were further stratified into neutrophils ($Ly6G^+$), and monocytes ($Ly6C^+$). (B) Identification of $CD3^+$ T cells, which were further characterized into $CD4^+$ and $CD8^+$ T lymphocyte subsets.

Supplementary Table S1 Nucleotide and amino acid variation sites in the EV-D68 parental strain and mouse-adapted strains

Position	VP2		VP3		VP3		VP3		VP1		2B		2C		3C		3D	
	nt 163	aa 55	nt 317	aa 106	nt 699	aa 234	nt 974	aa 325	nt 270	aa 90	nt 247	aa 83	nt 634	aa 212	nt 155	aa 52	nt 945	aa 315
P0	C	Q	T	Y	A	R	T	K	A	N	T	L	C	L	T	V	C	D
P1	·	·	·	·	·	·	·	·	·	N	·	·	·	·	·	·	·	·
P2	A	K	·	·	·	·	·	·	·	N	·	·	·	·	·	·	·	·
P3	A	K	·	·	·	·	·	·	·	N	·	·	·	·	·	·	·	·
P4	A	K	·	·	·	·	A	·	G	K	·	·	·	·	·	·	·	·
P5	A	K	C	·	G	G	A	·	G	K	·	·	T	·	C	A	·	·
P6	A	K	C	·	G	G	A	·	G	K	C	·	T	·	C	A	·	·
P7	A	K	C	·	G	G	A	·	G	K	C	·	T	·	C	A	·	·
P8	A	K	C	·	G	G	A	·	G	K	C	·	T	·	C	A	T	·
P9	A	K	C	·	G	G	A	·	G	K	C	·	T	·	C	A	T	·

Note: The shaded area indicates the only site in the VP1 region where amino acid mutations occur during the passaging process
